# Comprehensive Evaluation of *Colocasia affinis* Schott Leaf Extracts: Anti-Inflammatory, Antioxidant, Antimicrobial, and Thrombolytic Activities Alongside Molecular Docking Studies

**DOI:** 10.1155/sci5/5115015

**Published:** 2025-11-11

**Authors:** Mohammed Fazlul Karim, Mohammad Arman, Syed Mohammed Tareq, Shahidul Islam, Sourav Kumar Shil, Md. Hassan Kawsar, Mohammad Nazmul Islam

**Affiliations:** ^1^Department of Pharmacy, Southern University of Bangladesh, Chittagong, Bangladesh; ^2^Department of Pharmacy, International Islamic University Chittagong, Chittagong 4318, Bangladesh; ^3^Department of Pharmacy, State University of Bangladesh, Dhaka 1205, Bangladesh; ^4^Department of Pharmacy, Jahangirnagar University, Savar, Dhaka 1342, Bangladesh

**Keywords:** anti-inflammatory, antimicrobial, antioxidant, *Colocasia affinis*, phytochemical, thrombolytic

## Abstract

There has been a recent rise in the utilization of complementary herbal medicines as a means to discover efficacious alternative treatments that mitigate the negative consequences of pharmaceuticals. *Colocasia affinis* Schott is a member of the Araceae family, with various components such as the root, fruit, and leaves utilized for medicinal purposes. This study aims to explore the in vitro phytochemical, antioxidant, antimicrobial, anti-inflammatory, and thrombolytic properties of the ethanolic extract obtained from the leaves of *C. affinis* (EECA) using an experimental approach. The extract derived from EECA revealed the presence of secondary metabolites, including alkaloids, glycosides, flavonoids, tannins, steroids, saponins, carbohydrates, amides, phenols, and reducing sugars, and demonstrated remarkable antioxidant activity in the DPPH scavenging assay (IC_50_ = 60.36 μg/mL). Secondly, five clinical isolates of bacteria, namely, *Escherichia coli, Salmonella paratyphi, Salmonella typhi, Pseudomonas aeruginosa,* and *Vibrio cholerae*, were employed to examine the antimicrobial properties of EECA. In the context of thrombolytic activity, EECA demonstrated a noteworthy level of clot lysis (39.086 ± 0.570% and 23.111 ± 0.398%) at doses of 500 and 250 μg/mL, respectively, when compared to streptokinase. Furthermore, EECA exhibited a significant anti-inflammatory effect, as evidenced by the inhibition of protein denaturation (60.24 ± 1.49, 43.81 ± 1.76, and 30.83 ± 2.57) across concentration ranges of 500, 250, and 125 μg/mL. The findings indicated the presence of phytochemicals and notable free radical scavenging activity. EECA exhibited a broad range of antimicrobial activity, along with notable thrombolytic and anti-inflammatory effects. This investigation presents empirical evidence that supports the application of EECA in traditional medicine.

## 1. Introduction

The bounty of nature has bestowed upon us an abundant reservoir of remedies for every ailment that afflicts humanity. Due to mankind's inherent inquisitiveness, the accumulation of medicinal knowledge has spanned millennia, leading to the development of a multitude of effective methods to secure healthcare [1]. Many well-known drugs from the 20th century were derived from prehistoric medicinal practices that utilized particular plants to treat various health issues. Large-scale facilities currently synthesize several plant components for application in medicinal formulations [2]. About 25 to 30% of all current medications are thought to be obtained either directly or indirectly from higher plants. The herbal goods industry is made up of a number of interconnected subsectors, including culinary herbs and spices, functional foods, nutraceuticals, phytochemicals, flavors and perfumes, and herbal tea [3, 4]. Approximately 50% of all exports of Indian botanical goods go to the United States, which is currently their greatest market. The top four countries for herbal drug imports are Japan, Hong Kong, Korea, and Singapore, accounting for 66% of China's exports of botanical drugs. Botanical medicine holds a significant market share for pharmaceuticals in the European Union [5]. The current time has focused on the analysis of phytoconstituents, which are potential candidates for pharmaceutical applications, as well as checking medical plants and seeds for other drugs. Researchers have looked at and exploited phytoconstituents that naturally occur in plants, such as tocopherols, carotenoids, ascorbates, polyphenolics, and terpenoids, as alternative medicines to treat a wide range of conditions induced by oxidative stress [6]. Phenolic compounds exhibit a range of biological features that are advantageous to human health, such as anti-inflammatory, anticancer, antimicrobial, and antiallergic effects [7].

Atherosclerosis, thrombosis, plaque rupture, myocardial injury, and failure are all caused by oxidative stress–induced inflammation, which are all symptoms of significant cardiac disorders in chronic situations [8]. Research has shown that inflammation is involved in the majority of immune-related conditions, including infectious diseases induced by pathogens, allergies, malignancies, and autoimmune disorders, despite its role as the body's natural protective mechanism [9, 10]. In order to stop immune cells from doing additional damage to tissues and to stop the development of inflammation-related chronic diseases, it is crucial to regulate inflammatory responses. When these features are present, anti-inflammatory medications with fewer side effects are more appealing. There are many things in tea, like arginine, flavonoids, glutathione, selenium, taurine, and vitamins C, E, and A, that are antioxidants. They protect cells from damage caused by free radicals. Many synthetic medications used to treat these conditions have unwanted side effects and do not work well enough to be considered effective treatments. The development of drugs that can effectively neutralize reactive oxygen and nitrogen species (RONS), alleviate inflammation, exhibit thrombolytic activity, and possess minimal adverse effects would represent a major breakthrough in pharmaceutical research. Plants engage in secondary metabolism to generate a diverse array of phytochemicals, which possess various biological roles, including those with notable medicinal efficacy [11].

The development of blood clots has been a serious issue with blood circulation. By obstructing the blood vessel, thrombus or emboli prevent regular blood flow and oxygen delivery to tissues [12]. The tissue in that location becomes necrotic as a result. Streptokinase is a notable and often used thrombolytic drug for the treatment of myocardial infarction. It is important to note that all currently available thrombolytic drugs possess significant limitations, including the necessity for high doses to achieve optimal efficacy, a limited capacity to selectively target specific fibrin types, and a considerable risk of inducing bleeding. Therefore, it is necessary to develop natural alternatives to synthetic thrombolytic medications that are safer, more cost-effective, and easily available which should have a wide range of modes of action and fewer adverse effects.

The widespread application of antimicrobial agents in the health of people, agriculture, and veterinary medicine presents considerable concerns, as their indiscriminate use has led to the emergence of resistance in pathogens. The demand for the development of new antimicrobial drugs is currently significant due to the limited availability of antibiotic medications. It has been discovered that the artificial antibiotics could have negative effects on the body. Finding new antimicrobials derived from natural items has followed as a result [13].

**Figure 1:**
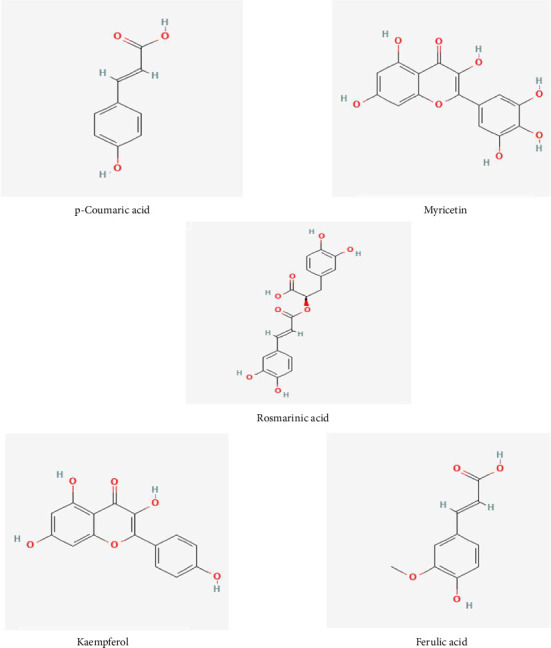
Chemical structures of the potential antioxidant, thrombolytic, and anti-inflammatory compounds screened from *C. affinis* leaves via computational analysis: p-coumaric acid, trans-ferulic acid, rosmarinic acid, myricetin, and kaempferol.

Much is unknown about the possibility of higher plants as a source of new medicinal compounds. Only a tiny number of the estimated 250,000 to 500,000 plant species have been evaluated for phytochemicals, and an even smaller number have been investigated for biological or pharmacological activity [14]. Phytochemical extraction of *Colocasia affinis* Schott leaves revealed the presence of many separate chemicals, including *p*-coumaric acid, myricetin, trans-ferulic acid, rosmarinic acid, and kaempferol [15]. As a result, in silico investigations were carried out based on the specified literature and availability (Table 1 and Figure 1). The utilization of in vitro and in silico models offers valuable insights into the effectiveness of plants in treating diverse diseases, as well as the characteristics of the plants for future investigation. On the other hand, no antioxidant, antimicrobial, anti-inflammatory, and thrombolytic activity investigations of EECA leaves (ethanolic extract of *C. affinis)* have been conducted using modern pharmacological methods yet. Taking all of this into consideration, in vitro and computational evaluation is being carried out to assess the anti-inflammatory, antibacterial, thrombolytic, and antioxidant properties of *C. affinis* ethanol extract.

## 2. Materials and Methods

### 2.1. Drugs and Chemicals

The Square Pharmaceuticals Ltd, Bangladesh, supplied a vial of lyophilized streptokinase (1,500,000 IU), along with ascorbic acid and ciprofloxacin. The chemicals ethanol (80% concentration), acetic anhydride, potassium iodide, sulfuric acid, acetylsalicylic acid, and sodium hydroxide (standard concentrations) were obtained from Merck (Darmstadt, Germany). Hindon India Pvt Ltd. supplied the chloroform and ferric chloride (5% w/v). Sigma Chemicals Co. of St. Louis, MO, supplied the glacial acetic acid.

### 2.2. Plant Material Collection and Identification and Extract Preparation (EECA)

*Colocasia affinis* (Family: Araceae) was selected for its medicinal properties. Taxonomic identification was performed by Mr. Md. Saidul Alam, Senior Researcher at the Forest Research Institute (BFRI) Herbarium, Chattogram, Bangladesh. Mature leaves were collected from the hill tracts of Rangamati, Chattogram, in September during peak growth. The leaves were air-dried in a cool, shaded area for 16 days, and then ground into a coarse powder using a laboratory grinder. The powdered material was stored in airtight containers in a cool, dry, and dark environment until extraction. Approximately 300 g of powdered leaves were subjected to Soxhlet extraction using 80% v/v ethanol (1.3 L) over seven consecutive cycles to ensure exhaustive extraction of phytochemicals. The solvent was removed under reduced pressure using a rotavapor (Buchi Flawil, Switzerland) to yield the concentrated crude ethanol extract [16]. The extract was stored at 4°C until further analysis.

### 2.3. Phytochemical Analysis

A stock solution of EECA was prepared by dissolving 1 g of extract in 100 mL of 80% ethanol. Qualitative phytochemical analysis was performed according to standard procedures [17, 18] in order to detect the existence of phytoconstituents such as alkaloids (Mayer's and Dragendorff's tests), flavonoids (alkaline reagent test), carbohydrates (Molisch's test), saponins (froth formation test), tannins (ferric chloride [5% w/v] test), glycosides (Keller–Kiliani test for cardiac glycosides and Borntrager's test for anthraquinone glycosides), reducing sugars (Fehling's test), steroids (Liebermann–Burchard test using acetic anhydride and sulfuric acid), phenols (Fehling's test), and gums (ethanol precipitation method). All tests were performed in triplicate to ensure reproducibility.

### 2.4. In Vitro Antioxidant Activity Using 2,2-Diphenyl-1-picrylhydrazyl (DPPH) Assay

The antioxidant potential of EECA was evaluated using the DPPH free radical scavenging assay. Serial dilutions of EECA were prepared at concentrations of 100, 80, 60, 40, 20, and 10 μg/mL. Negative control: 95% ethanol; Positive control: Ascorbic acid. Each extract solution (1 mL) was mixed with an equal volume of 0.1 mM DPPH solution in ethanol and incubated in the dark at room temperature for 30 min. Absorbance was measured at 517 nm using a UV-Vis spectrophotometer. The half maximum inhibitory concentration (IC_50_) was determined graphically in comparison to standard ascorbic acid [19] and the percentage of the DPPH free radical was calculated using the following equation:

(1) $$\begin{array}{c} \%\text{ Scavenging activity}=\frac{\text{Absorbance of control}-\text{Absorbance of test sample}}{\text{Absorbance of control}}\times 100. \end{array}$$

### 2.5. In Vitro Antimicrobial Activity by Disc Diffusion Assay

Agar well diffusion tests were used to conduct the antibacterial testing. Five clinical isolates of bacteria, including *Escherichia coli*, *Salmonella paratyphi*, *Salmonella typhi*, *Pseudomonas aeruginosa*, and *Vibrio cholerae*, were used to investigate the antimicrobial properties of EECA. The results were compared to ciprofloxacin as a standard [20]. The antibacterial activity of the plant extracts was evaluated by measuring the zone of inhibition (in mm) against five pathogenic microorganisms. This was done by comparing the percentage of inhibition exhibited by the test groups to that of the standard antibiotic ciprofloxacin (30 μg/disc) and the negative control.

### 2.6. In Vitro Thrombolytic Activity

Five milliliters of venous blood were collected from each research participant and evenly distributed into five preweighed tubes, with 500 μL of blood allocated per tube. The tubes were then incubated at 37°C. During incubation, the samples were treated with streptokinase as a positive control, to evaluate the thrombolytic potential of the EECA extract. Once clot formation occurred, the serum was carefully and permanently removed to prevent any interference with the clots. Each tube containing the clot was then weighed twice to accurately determine the clot [21]. The following chart illustrates how the discrepancies between weights obtained before and after clot lysis were reported as a percentage of clot lysis:

(2) $$\begin{array}{c} \%\text{ of clot lysis}=\left(\frac{\text{wt of released clot}}{\text{clot wt}}\right)\times 100. \end{array}$$

### 2.7. In Vitro Anti-Inflammatory Assay by Protein Denaturation

Through slight adjustments, Sakat et al. [22] did study the anti-inflammatory potential of EECA. The reaction mixture consisted of 3 mL of a 5% solution of egg albumin and 3 mL of test extracts with varying concentrations. The pH of the test extracts was modified to a target value of 5.6 ± 0.2 by adding 0.1 N HCl. This resulted in final concentration levels of 125, 250, and 500 μg/mL. For negative control purposes, an equal amount of ethanol was utilized, and diclofenac sodium was used as positive control. The solutions were regularly heated at 57°C for 20 min following a 15-minute incubation at 37 2°C in a biochemical oxygen demand (BOD) incubator. The absorbance at a wavelength of 660 nm was subsequently determined using a blank vehicle [23]. According to the following formula, the percentage of protein denaturation suppression was calculated:

(3) $$\begin{array}{c} \%\text{ Inhibition}=\frac{\text{Absorbance of control}-\text{Absorbance of test sample}}{\text{Absorbance of control}}\times 100. \end{array}$$

### 2.8. Computer-Aided Approach to Molecular Docking Analysis

#### 2.8.1. Ligand and Protein Preparations

Five distinct 3D molecular structures of isolates originating from the EECA region were retrieved in SDF file format from the PubChem database. These structures were imported into the Schrödinger Suite Maestro (Version 11.1) and prepared using the LigPrep module, applying the OPLS-2005 force field for geometry optimization. During ligand preparation, ionization states were generated at pH 7.0 ± 2.0 using Epik, stereoisomers were retained, and tautomeric forms were minimized to ensure biologically relevant conformations. Additionally, the three-dimensional crystal structure of the target enzyme was obtained from the Protein Data Bank (PDB) maintained by the Structural Bioinformatics community [24]. The protein was selected based on resolution (< 2.5 Å), mutation, expression system, methods of expression, availability of a co-crystallized ligand, and biological relevance to the pathway of interest. Protein preparation was carried out in the Protein Preparation Wizard of Schrödinger which included adding hydrogen atoms, assigning bond orders, optimizing side chains, and minimizing the structure with the OPLS-2005 force field. Water molecules beyond 5 Å from the active site were removed to avoid steric interference. The proteins mentioned include human aldo-keto reductase family 1 member C3 (AKR1C3) with PDB IDs 1S1P and 1S1R, peroxisome proliferator-activated receptor gamma (PPAR gamma) with PDB ID 4XTA, tissue plasminogen activator (tPA) with PDB ID 1A5H, factor Xa with PDB ID 2BOK, and NADH oxidase with PDB IDs 1XHC and 2CDU. The molecular docking study was conducted using the Schrödinger-Maestro (v 11.1) software, employing the established method described in earlier studies [25].

#### 2.8.2. Receptor Grid Generation and Ligand Docking

The selected macromolecular proteins were positioned on a specific chain identified by PockDrug. The proteins were placed in a box with dimensions of 10 Å in the *X*, *Y*, and *Z* directions for the purpose of selecting a binding site using Receptor Grid Generation. The Schrödinger-Maestro version 11.1 SP software was utilized to perform flexible ligand docking, incorporating penalties for non-cis/trans amide bonds. The ultimate score, determined by energy-conserving positions, was computed utilizing Glide%. The optimal docking position with the lowest Glide score value was recorded for each ligand. The selected compounds were subjected to molecular docking studies to investigate their anti-inflammatory, thrombolytic, and antioxidant effects against various polymerases [26].

### 2.9. PASS Prediction Study

Using the PASS digital tools, these five isolated samples were further evaluated for antioxidant, anti-inflammatory, thrombolytic, and other biological properties. The prediction result is displayed as a list of activities with adequate probable activity (Pa) and probable inactivity (Pi) percentages, where Pa and Pi represent the estimated likelihood of a compound being biologically active or inactive, respectively [27].

### 2.10. In Silico ADME/T Study and Oral Toxicological Property Prediction

The pharmacokinetic properties of the selected compounds were evaluated using the SwissADME web server. Drug-likeness was initially assessed based on Lipinski's rule of five (molecular weight, LogP, hydrogen bond donors and acceptors, and molar refractivity) [28]. In silico toxicity of pigment-derived compounds was assessed using online tools like ProTox-II, which predict potential toxic effects such as hepatotoxicity, carcinogenicity, immunotoxicity, mutagenicity, and cytotoxicity [29]. This prediction was used to complement the ADME assessment and to highlight the potential therapeutic safety of the studied molecules.

### 2.11. Statistical Analysis

Data were expressed as mean ± standard error of the mean (SEM). Statistical analysis was performed using one-way ANOVA, followed by Dunnett's post hoc test for multiple group comparisons. For comparisons between two groups, Student's *t*-test was applied. Differences were considered statistically significant when the *p* value was less than 0.05.

## 3. Results

### 3.1. Qualitative Phytochemical Analysis

Phytochemical examination of the EECA revealed the presence of alkaloids, glycosides, flavonoids, tannins, steroids, saponins, carbohydrates, amides, phenols, and reducing sugars, which is shown in Table 2.

### 3.2. In Vitro Antioxidant Activity by DPPH Assay

**Figure 2:**
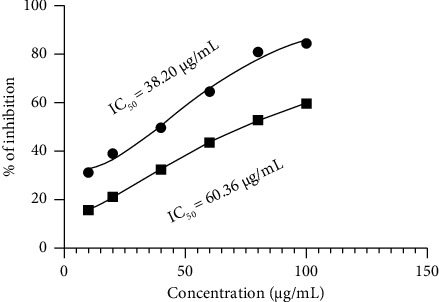
In vitro antioxidant effect of ethanol extracts of *C. affinis*.

The quantitative experiment revealed that the EECA exhibited the ability to neutralize free radicals in the DPPH method, yielding an IC_50_ value of 60.36 μg/mL. In comparison, ascorbic acid, a widely recognized antioxidant standard, demonstrated an IC_50_ value of 38.20 μg/mL (Figure 2).

### 3.3. In Vitro Antimicrobial Activity by Disc Diffusion Assay

The zone of inhibition for the EECA ranges from 6.3 to 8.5 mm, showing a modest inhibition against microbial growth. The more zone of reduction produced by the EECA was reported to be 8.5 ± 0.230 and 7.3 ± 0.416 mm against *S. paratyphi* and *P. aeruginosa*, respectively, while ciprofloxacin exhibited highest zone of inhibition against *V. cholerae* and *S. paratyphi* (22.23 ± 0.458 and 19.967 ± 0.448).  Table 3 shows the inhibition of microorganisms.

### 3.4. In Vitro Thrombolytic Activity

**Figure 3:**
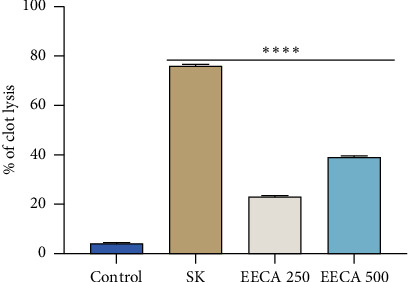
In vitro thrombolytic effect of ethanol extracts of *C. affinis* leaves. Values are expressed as mean ± SEM (*n* = 5); ^∗∗∗∗^*p* < 0.0001 are statistically significant compared to control followed by Dunnett test (GraphPad Prism 8.4). EECA = ethanol extract of *Colocasia affinis*.

In Figure 3, the thrombolytic activity of EECA is displayed. In contrast to streptokinase, the extracts showed clot lysis of 39.086 ± 0.570% and 23.111 ± 0.398% at doses 500 and 250 μg/mL compared to 76.046 ± 0.683% for streptokinase (positive control). The mean difference in clot lysis percentage compared to the negative control (sterile distilled water) showed statistically significant results (^∗∗∗∗^*p* < 0.0001).

### 3.5. In Vitro Anti-Inflammatory Assay

**Figure 4:**
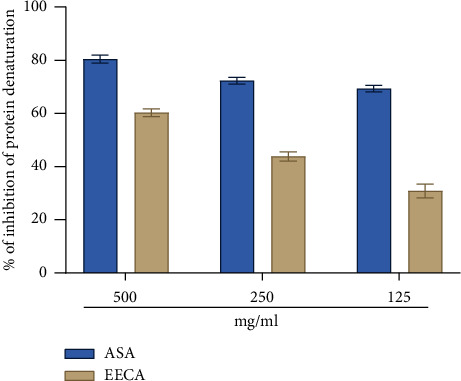
In vitro anti-inflammatory effects of ethanol extract of *C. affinis* leaves. Values are expressed as mean ± SEM (*n* = 5). EECA = ethanol extract of *Colocasia affinis*.

The overall inhibition of denaturation of protein was calculated by measuring the different treatment groups and converting these values to anti-inflammatory activity. According to the current research, EECA inhibits protein denaturation in a concentration-dependent manner. In comparison to the standard, the EECA had significant anti-inflammatory effect inhibition of protein denaturation (60.24 ± 1.49, 43.81 ± 1.76, and 30.83 ± 2.57) at concentration ranges of 500, 250, and 125 μg/mL (Figure 4).

### 3.6. Molecular Docking Analysis of Antioxidant Activity

**Figure 5:**
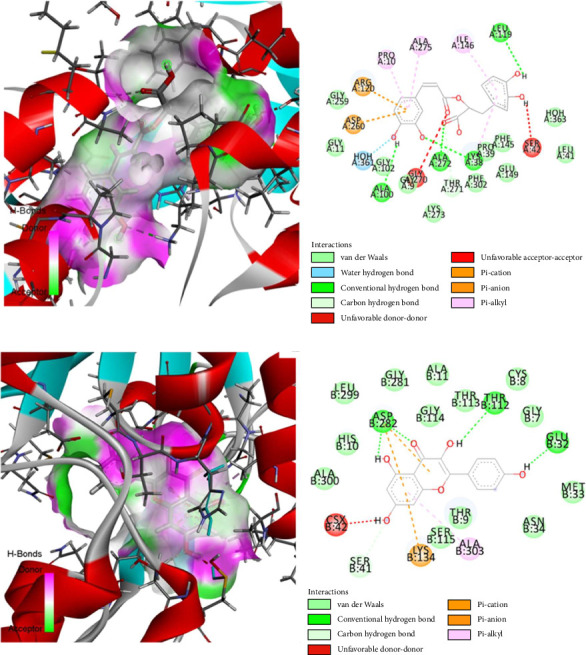
Molecular docking of (a) rosmarinic acid and (b) kaempferol with NADH oxidase (PDB ID: 1XH and 2CDU) enzymes for antioxidant activity.

The computational methodology was utilized to analyze the individual compounds, and the results obtained using Schrödinger-Maestro (Version 11.1) are presented in Table 4. The binding energies of rosmarinic acid, myricetin, and kaempferol with NADH oxidase (PDB ID: 1XH) were found to be −8.178, −6.733, and −6.465 kcal/mol, respectively. These compounds also exhibited binding affinities of −7.874, −6.664, and −7.109 kcal/mol with NADH oxidase (PDB ID: 2CDU), respectively, making them the most effective interactions. When, dextromethorphan revealed docking score −5.447 and −5.498 kcal/mol, the two most prominent poses of each protein have been preserved in pdbqt format and then assessed for protein-lead chemical interaction using the Discovery Studio 3.5 Visualizer. Two complexes were chosen based on the amount of interactions between the target proteins and chemicals, as shown in Figure 5.

### 3.7. Molecular Docking Analysis of Thrombolytic Activity

**Figure 6:**
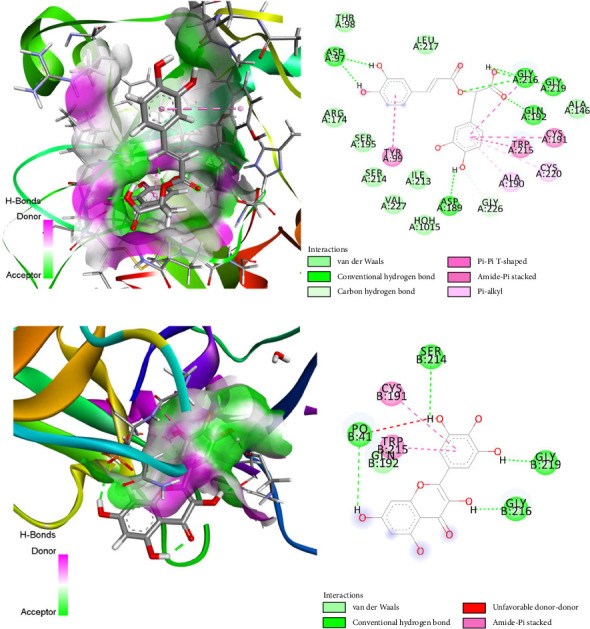
The best rank pose of thrombolytic activity of (a) rosmarinic acid and (b) kaempferol with inhibitory site of tPA (PDB ID: 1A5H and 1RTF) and factor Xa (PDB ID: 2BOK) enzymes for thrombolytic activity.

Table 5 and Figure 6 display the docking score and glide energy of the compounds, as well as the standard medication streptokinase. Meanwhile, Figure 6 illustrates the most significant interactions between the ligands and enzymes. Based on the table, it is evident that rosmarinic acid, myricetin, and kaempferol exhibited the highest docking scores of −7.368, −6.297, and −7.266 kcal/mol, respectively, with tPA (PDB ID: 1A5H and 1RTF). In addition, these compounds exhibited the most accurate docking scores (−6.396 and −6.17 kcal/mol) while interacting with the factor Xa enzymes (PDB ID: 2BOK), respectively. In addition, the conventional medication streptokinase is linked with tPA (PDB ID: 1A5H and 1RTF) and factor Xa (PDB ID: 2BOK) enzymes, which had docking scores of −6.173, −6.294, and −5.985 kcal/mol.

### 3.8. Molecular Docking Analysis of Anti-Inflammatory Activity

**Figure 7:**
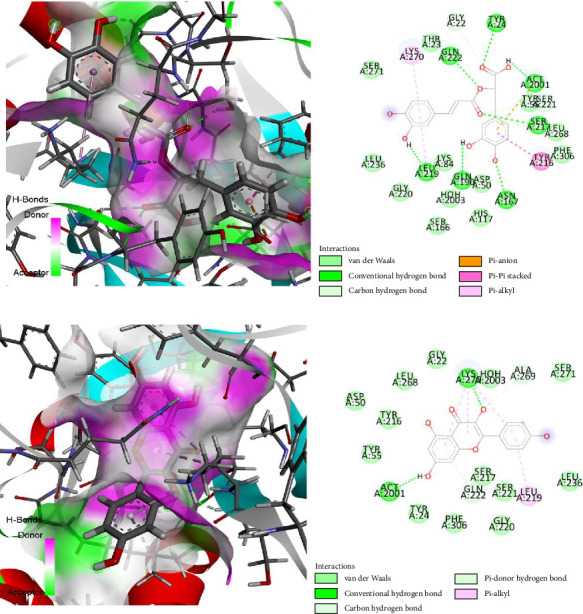
The best rank pose of the anti-inflammatory activity of (a) rosmarinic acid and (b) kaempferol in the binding pocket of AKR1C3 (PDB ID: 1S1P and 1S1R) and PPAR gamma (PDB ID: 4XTA) enzymes for anti-inflammatory activity.

Molecular docking investigation of anti-inflammatory properties was done using the Schrödinger-Maestro (v 11.1) for the five drugs with the human AKR1C3 and PPAR gamma (Table 6), whereas interaction of the ligands with receptor is presented in Figure 7. The docked binding mode was used to link the function of binding affinity (docking score), structural properties associated with these chemical compounds, and their biological activities against the AKR1C3 and PPAR gamma receptors. The experimental study was juxtaposed with computational efforts related with the presently prescribed drugs for the treatment of different anti-inflammatory agents, as well as substances used in preclinical and clinical studies. Rosmarinic acid and kaempferol have higher binding affinities to several enzymes than any other substances, registering values of −7.417 and −6.680 kcal/mol with AKR1C3 (PDB ID: 1S1R), while diclofenac sodium displayed a binding affinity of −5.90 kcal/mol. In the interim, these two compounds yielded −8.207 and −7.430 kcal/mol with (PDB ID: 4XTA), while diclofenac sodium exhibited −7.380 kcal/mol. The results presented here could act as a foundational basis for the structure-driven enhancement of these innovative pharmaceuticals, informed by receptor interactions and binding affinities.

### 3.9. PASS Prediction

Pa and Pi are statistical indicators of whether the substance is active or inactive. Only those types of activities for which Pa > Pi are likely to be exhibited by the compound. When Pa > 0.3, the compound is predicted to show that particular activity in experimental evaluations. However, the probability of this compound demonstrating the activity depends on its Pa value Table 7 shows a subset of the expected pharmacological activity for isolates of EECA.

### 3.10. In Silico ADME/T Study and Oral Toxicological Property Prediction

 These studies looked on the suitability of chemicals that might be employed as therapeutic molecules. This study examined several variables, including the molecular weight (less than 500 g/mol), the number of hydrogen bond acceptors (up to 10), the number of hydrogen bond donors (up to 5), the logP value (less than 5), and the molar refractivity range (40–130) of the compounds. These variables are detailed in Table 8 of the ADME/T profile instance using SwissADME. This suggests that all derivative substances under consideration have the potential to be exploited as medication candidates. The ProTox-II online programme was used again to estimate the toxicological qualities of the compounds, and the results are displayed in Table 9. some toxicity concerns, their drug-likeness supports further validation. These findings align with molecular docking results, highlighting their potential for QSAR modeling, homology studies, and experimental evaluation (Table 9).

## 4. Discussion

Nature is considered as the best source for medicines as a variety of natural products exist with promising medicinal values. The present investigation has been taken into consideration with an important ethnomedicine, *C. affinis*. The plant has the potential to provide multiple biological effects depending on the presence of different phytochemicals [30]. Numerous research studies have revealed that the flavonoids present in plant extracts have anti-inflammatory, antibacterial, and antioxidant effects [31]. Saponins, tannins, flavonoids, and glycosides contribute to anti-inflammatory effects [32], while alkaloids, saponins, tannins, flavonoids, and steroids are primarily responsible for antimicrobial activity [33]. As *C. affinis* indicated the presence of flavonoids, tannins, saponins, and other chemical components in the phytochemical test, it is possible that these chemical components are responsible for the plant's antioxidant, anti-inflammatory, antibacterial, and thrombolytic features.

The presence of a yellow spot against a purple background on the TLC plate in the DPPH assay indicated that the EECA possessed the capacity to eliminate free radicals in a qualitative antioxidant experiment using TLC. It has a quantifiable impact on the antioxidant activity. EECA exhibited significant free radical scavenging activity in the quantitative DPPH experiment, with an IC_50_ value of 60.36 μg/mL. Plant-derived antioxidants commonly have a phenolic component. Phenolic chemicals can easily transfer electrons to reactive radicals, hence inhibiting radical chain reactions, thanks to the resonance stability of the phenoxy radical [34].

Antimicrobial evaluation shows that the investigated extract has inhibitory effectiveness against microbes. The ethanol crude extract was discovered to have good inhibitory properties in this experiment against a number of pathogenic bacteria species related to the conventional antibiotic ciprofloxacin. This characteristic shows the existence of one or more chemical molecules with antibacterial properties in the crude extract. However, more investigation into this plant is required to locate and isolate the chemicals that are accountable for each of their unique mechanisms of action.

The critical occurrence of thrombosis, or the creation of blood clots, occurs when platelets, tissue factor, and fibrin are deposited to seal the injured areas of the endothelial cell surface or blood artery [35]. Various thrombolytic medicines are used to dissolve blood clots that have already formed in blood arteries; however, these medications have drawbacks and can have negative, even fatal, effects [36, 37]. Numerous studies have been done to identify plants, natural foods, and their supplements that have an antithrombotic impact, and there is evidence that eating these foods helps to avoid heart attacks and strokes [38, 39]. In light of the results of the current investigation, it can be said that the various leaf extracts of *C. affinis* have the ability to dissolve clots by severing the fibrinogen and fibrin that are present within a clot. Additionally, the plant extract may contain bioactive chemicals, which may support continuing cardiovascular medication discovery from floristic resources, according to a positive result in a test for thrombolytic activity.

Anti-inflammatory properties have a preliminary relationship with membrane stabilizing activity [40]. Many diseases emerge when phagocytes discharge hydrolytic components into the extracellular area during inflammatory processes. The in vitro anti-inflammatory activity of pharmaceuticals or plant extracts can be determined by using hypotonic solution and heat-induced erythrocyte membrane lysis, which stabilizes the erythrocyte membrane. The membranes of red blood cells undergo lysis, leading to hemolysis, oxidation of hemoglobin, and subsequent interactions with harmful agents such as hypotonic solutions, elevated temperatures, and methyl salicylate [41, 42]. Membrane stabilization prevents the flow of fluids and serum proteins into the tissues during a time when inflammatory mediators have higher permeability [43]. The current analysis demonstrated a significant difference in the percentage of hemolysis inhibition between the standard and tested extracts (*p* < 0.05). Research has indicated that tannin and saponins can bind to cations, resulting in the stabilization of the erythrocyte membrane. Additionally, investigations have revealed that flavonoids have a stabilizing impact on lysosomes [44].

EECA was analyzed using computer-assisted drug discovery systems to explore its antioxidant, thrombolytic, and anti-inflammatory properties. Bioactive compounds were selected based on GC-MS analysis and literature review [45], followed by PASS prediction and ADME/T evaluation. Isolates that complied with Lipinski's “Rule of Five” were further analyzed using molecular docking to predict possible interactions with target proteins.

Molecular docking is an effective computational approach used in structural molecular biology and computer-assisted drug design. It predicts the interactions between ligands and targets, enabling researchers to understand how active isolates bind to key enzymes [46]. The identified protein targets play essential roles in a range of pharmacological processes, encompassing antioxidant, anti-inflammatory, and thrombolytic activities. The evaluation of the antioxidant activity of the isolated compounds was conducted through a computational methodology, utilizing the Schrödinger-Maestro (Version 11.1) software. The findings acquired are delineated in Table 4. The interactions of rosmarinic acid, myricetin, and kaempferol with NADH oxidase (PDB ID: 1XH) were assessed by evaluating their binding energy, revealing the most effective combinations. Among the various interactions studied, the binding affinity with NADH oxidase (PDB ID: 2CDU) emerged as the most promising. Based on the docking scores, these three compounds may be associated with a possible antidepressant effect and are therefore recommended for further investigation using QSAR and homology modeling. In addition, rosmarinic acid and kaempferol exhibited superior anti-inflammatory effects compared to diclofenac sodium when interacting with AKR1C3 (PDB ID: 1S1P). Furthermore, trans-ferulic acid, rosmarinic acid, and kaempferol showed the highest binding affinity. Compounds that contain rosmarinic acid and kaempferol had the highest docking score with PPAR gamma (PDB ID: 4XTA). These findings have the potential to facilitate the development of novel drugs by optimizing their structure through receptor interactions and binding scores. In addition, rosmarinic acid, myricetin, and kaempferol have demonstrated possible thrombolytic activity with tPA (PDB ID: 1A5H and 1RTF) and factor Xa (PDB ID: 2BOK). Nevertheless, the three compounds mentioned above exhibited superior performance compared to the commercially accessible drug streptokinase. These findings, in combination with in vitro and in silico analyses, highlight the possible pharmacological relevance of EECA and its bioactive constituents, providing a foundation for further in-depth experimental validation, including in vivo studies and molecular dynamics simulations.

However, the PASS prediction techniques were developed on a dataset of 20,000 main compounds [47] and over 4000 different biological activities based on a structural formula with an average accuracy of roughly 90% [48]. The PASS algorithm utilized structure-activity relationship (SAR) analysis of a training set consisting of many compounds with diverse biological activities to forecast potential biological effects of plant isolates [49]. Before beginning the inquiry, the PASS program was used to determine if plant isolates, using the SAR technique, meet the requirements of the training set in the PASS database.

The isolates were evaluated for adherence to Lipinski's “Rule of Five” concerning bioavailability. The findings demonstrate that the examined isolates conform to the “Rule of Five,” indicating potential drug-like properties in relation to oxidation, inflammation, and thrombosis formation. Lipinski's five principles stipulate that any drug or chemical designed for oral use must meet specific criteria. If any substances violate the criteria, their bioavailability may be compromised. Based on the results of this inquiry, the total number of infractions falls between the ranges of 0–1. Theoretically, the results indicate that all isolates conform to this standard and are promising candidates for therapy due to their high bioavailability. This study conducted a detailed analysis of the toxicological characteristics of the selected isolates using ProTox-II online tools. The results showed that the majority of the compounds did not exhibit any harmful effects on the liver, did not have the potential to cause cancer, did not affect the immune system, did not have mutagenic properties, and did not show any toxicity to cells.

Although the present study highlights the pharmacological potential of EECA and its bioactive isolates, several limitations should be acknowledged including full isolation, absence of molecular dynamics simulations, and restriction to in vitro and in silico analyses. In vivo studies and clinical trials are needed to confirm efficacy, pharmacokinetics, safety, and therapeutic potential. Addressing these gaps will strengthen the translational value of EECA as a medicinal agent.

## 5. Conclusion

The current study has shown that the ethanol extract of *C. affinis* may have antioxidant, anti-inflammatory, and thrombolytic effects. These effects are likely related to the existence of secondary metabolites that have promising pharmacological properties. However, the antimicrobial evaluation demonstrated the notable antibiotic action of EECA. In the context of a computer-assisted analysis, all chemicals collected from the published literature satisfy Lipinski's rule of five. Among these, rosmarinic acid and kaempferol exhibit the highest spontaneous binding energy for antioxidant, anti-inflammatory, and thrombolytic properties. The ADME/T analysis of the extracted bioactive compounds demonstrated a good level of safety and the potential for oral bioavailability from a drug development perspective. Nevertheless, it is highly advisable to do a more thorough investigation of the extract and fractions, including the process of isolating and identifying the main components, in order to definitively establish the specific bioactive compounds responsible for the mentioned pharmacological qualities. Conducting thorough investigations is also advised to clarify the potential mechanisms of action in the animal model and then in humans to determine clinical effectiveness.

## Figures and Tables

**Table 1:** Quantitative compounds retrieved from literature review of *C. affinis*.

Compound name	Solvents and plant parts	Molecular formula	Molecular weight (g/mol)	Compound CID	Phytochemical class	References
p-Coumaric acid	Ethanol and leaves	C_9_H_8_O_3_	164.16	637542	Phenolic	[15]
trans-Ferulic acid		C_10_H_10_O_4_	194.18	445858	Phenolic	
Rosmarinic acid		C_18_H_16_O_8_	360.3	5281792	Phenolic	
Myricetin		C_15_H_10_O_8_	318.23	5281672	Flavonoid	
Kaempferol		C_15_H_10_O_6_	286.24	5280863	Flavonoid	

**Table 2:** Qualitative phytochemical screening of ethanol extracts of *C. affinis* leaves.

Phytochemicals	Leaf extract
Alkaloids	+
Glycosides	+
Steroids	+
Tannins	+
Flavonoids	+
Saponins	+
Carbohydrates	+
Reducing sugars	+
Gums	−
Amides	+
Phenols	+

*Note:* The presence of a phytochemical group is indicated by (+) and the absence is indicated by (−).

**Table 3:** Zones of inhibition by the treatment groups of EECA.

Microorganisms	Zone of inhibition	
	EECA (500 μg/disc)	Ciprofloxacin (30 μg/disc)
*P. aeruginosa*	7.3 ± 0.42	18.6 ± 0.27
*E. coli*	7.2 ± 0.21	17.5 ± 0.21
*S. paratyphi*	8.5 ± 0.23	20.0 ± 0.45
*S. typhi*	6.8 ± 0.18	15.7 ± 0.23
*V. cholerae*	6.8 ± 0.23	22.3 ± 0.46

**Table 4:** Binding energy (kcal/mol) of the isolates of EECA with NADH oxidase (PDB ID: 1XH and 2CDU) enzymes for antioxidant activity.

Compounds	PDB ID	
	1XHC	2CDU
Dextromethorphan	−5.447	−5.498
p-Coumaric acid	−5.938	−4.655
trans-Ferulic acid	−5.953	−5.742
Rosmarinic acid	−8.178	−7.874
Myricetin	−6.733	−6.664
Kaempferol	−6.465	−7.109

**Table 5:** Docking score (kcal/mol) of the isolates of EECA with tPA (PDB ID: 1A5H and 1RTF) and factor Xa (PDB ID: 2BOK) enzymes for thrombolytic activity.

Compounds	PDB ID		
	1A5H	1RTF	2BOK
Streptokinase	−6.173	−6.294	−6.485
p-Coumaric acid	−4.778	−6.068	−6.002
trans-Ferulic acid	−5.923	−6.171	−6.457
Rosmarinic acid	−7.368	−6.658	−7.77
Myricetin	−6.297	−6.502	−6.812
Kaempferol	−7.266	−6.501	−7.339

**Table 6:** Docking score (kcal/mol) of the major isolates of EECA with AKR1C3 (PDB ID: 1S1P and 1S1R) and PPAR gamma (PDB ID: 4XTA) enzymes for anti-inflammatory activity.

Compounds	PDB ID		
	1S1P	1S1R	4XTA
Diclofenac sodium	−6.059	−5.901	−7.380
p-Coumaric acid	−5.526	−5.352	−5.032
trans-Ferulic acid	−6.409	−5.668	−5.298
Rosmarinic acid	−7.149	−7.417	−8.207
Myricetin	−4.482	−5.515	−7.298
Kaempferol	−6.983	−6.680	−7.430

**Table 7:** Pharmacological activities predicted for the major isolates of EECA.

Compounds	Biological activity	Pa	Pi
p-Coumaric acid	Antioxidant	0.553	0.005
	Antimicrobial	—	—
	Anti-inflammatory	0.684	0.003
	Thrombolytic	0.541	0.023

trans-Ferulic acid (FA)	Antioxidant	0.540	0.005
	Antimicrobial	—	—
	Anti-inflammatory	0.661	0.003
	Thrombolytic	0.538	0.024

Rosmarinic acid	Antioxidant	0.539	0.005
	Antimicrobial	—	—
	Anti-inflammatory	0.496	0.008
	Thrombolytic	0.337	0.005

Myricetin	Antioxidant	0.924	0.003
	Antimicrobial	—	—
	Anti-inflammatory	0.720	0.013
	Thrombolytic	0.241	0.031

Kaempferol	Antioxidant	0.856	0.003
	Antimicrobial	—	—
	Anti-inflammatory	0.676	0.019
	Thrombolytic	0.461	0.053

**Table 8:** Physicochemical properties of the major isolates of EECA for good oral bioavailability.

Compounds	MW	HBA	HBD	Log *p*	AMR	Lipinski's violations
Rule	< 500 g/mol	≤ 10	≤ 5	≤ 5	40–130	≤ 1
p-Coumaric acid	164.16	3	2	0.95	45.13	0
trans-Ferulic acid	194.18	4	2	1.62	51.63	0
Rosmarinic acid	360.31	8	5	1.17	91.40	0
Myricetin	318.24	8	6	1.08	80.06	1
Kaempferol	286.24	6	4	1.70	76.01	0

**Table 9:** Toxicological properties predicted for the isolates of EECA.

Compounds	Prediction/probability				
	Hepatotoxicity	Carcinogenicity	Immunotoxicity	Mutagenicity	Cytotoxicity
p-Coumaric acid	Inactive/0.51	Active/0.50	Inactive/0.91	Inactive/0.93	Inactive/0.83
trans-Ferulic acid	Inactive/0.51	Inactive/0.61	Active/0.91	Inactive/0.96	Inactive/0.88
Rosmarinic acid	Active/0.69	Inactive/0.62	Active/0.96	Inactive/0.97	Inactive/0.93
Myricetin	Inactive/0.69	Active/0.68	Inactive/0.86	Active/0.51	Inactive/0.99
Kaempferol	Inactive/0.68	Inactive/0.72	Inactive/0.96	Inactive/0.52	Inactive/0.98

## Data Availability

All the data related to the manuscript have been mentioned.
